# The association between workload and quality of work life of nurses taking care of patients with COVID-19

**DOI:** 10.1186/s12912-023-01395-6

**Published:** 2023-07-07

**Authors:** Hassan Babamohamadi, Hossein Davari, Abbas-Ali Safari, Seifollah Alaei, Sajjad Rahimi Pordanjani

**Affiliations:** 1 Nursing Care Research Center, Education and Research Campus, Semnan University of Medical Sciences, Po Box: 3513138111, 5 Kilometer of Damghan Road, Semnan, Iran; 2 Department of Nursing, School of Nursing and Midwifery, Semnan University of Medical Sciences, Semnan, Iran; 3 Student Research Committee, Semnan University of Medical Sciences, Semnan, Iran; 4 Social Determinants of Health Research Center, Semnan University of Medical Sciences, Semnan, Iran; 5 Department of Community Medicine, School of Medicine, Semnan University of Medical Sciences, Semnan, Iran

**Keywords:** Workload, Quality of Work Life, Nurse, COVID-19

## Abstract

**Background:**

The COVID-19 epidemic has brought significant changes and complexities to nurses’ working conditions. Given the crucial role of health workers, particularly nurses, in providing healthcare services, it is essential to determine the nurses’ workload, and its association with the quality of work life (QWL) during COVID-19 epidemic, and to explain the factors predicting their QWL.

**Methods:**

A total of 250 nurses, who provided care for patients with COVID-19 in Imam Hossein Hospital of Shahrud, and met the inclusion criteria, were considered the samples in the present cross-sectional study in 2021–2022. Data were collected using the demographic questionnaire, NASA Task Load Index (TLX), and Walton’s QWL questionnaire, which were analyzed using SPSS26 and based on descriptive and inferential statistical tests. A p-value less than 0.05 was considered significant for all cases.

**Results:**

The nurses’ mean scores of workload and QWL were 71.43 ± 14.15 and 88.26 ± 19.5, respectively. Pearson’s correlation test indicated a significant inverse relationship between workload and QWL (*r=-0.308, p < 0.001*). The subscales with the highest perceived workload scores were physical demand and mental demand (14.82 ± 8.27; 14.36 ± 7.43), respectively, and the subscale with the lowest workload was overall performance (6.63 ± 6.31). The subscales with the highest scores for QWL were safety and health in working conditions and opportunity to use and develop human capabilities (15.46 ± 4.11; 14.52 ± 3.84), respectively. The subscales with the lowest scores were adequate and fair compensation, work and total living space (7.46 ± 2.38; 6.52 ± 2.47), respectively. The number of children (β = 4.61, *p* = 0.004), work experience (β= -0.54, *p* = 0.019), effort (β = 0.37, *p* = 0.033) and total workload (β= -0.44, *p* = 0.000) explained 13% of the variance of nurses’ QWL.

**Conclusions:**

The study’s findings showed that a higher workload score is associated with nurses’ lower perception of QWL. In order to improve the QWL of nurses, reducing the physical and mental demands of their workload and strengthening overall performance is necessary. Additionally, when promoting QWL, adequate and fair compensation and the work and living space should be considered. The researchers suggest that hospital managers should make more significant efforts to develop and promote the QWL of nurses. To achieve this goal, organizations can pay attention to other influential factors, primarily by increasing organizational support.

## Background

Nurses comprise the most significant healthcare and treatment systems workforce, serving as the care team’s backbone [1]. The World Health Organization (WHO) states that there are approximately 27 million nurses worldwide, accounting for 50% of all health workers, and projects that this number will increase by 9 million by 2030 [2]. Nurses are responsible for most care and treatment measures and often must take on additional tasks beyond their primary roles [3, 4]. Numerous studies have documented the high workload experienced by nurses [5, 6]. Furthermore, nurses face various stressors, including unhealthy work environments, continuous fatigue, challenging workplace relationships, occupational hazards, and demanding workloads that can negatively impact their professional performance [7]. Over the past few decades, research has highlighted the stressful and demanding nature of nursing, characterized by its specialization, complexity, and the need to manage emergencies [8]. Considering the interconnectedness between caregivers and care, it is essential to prioritize the quality of care and the satisfaction of the care providers [9].

Nurses are one of the most crucial pillars of healthcare organizations in various situations, including the COVID-19 pandemic [10]. Although the severity of COVID-19 is gradually decreasing, nurses have been providing care to patients in various sectors of hospitals, including emergency departments, intensive care units, and wards, for nearly three years. One study has even suggested that 80% of the workload related to patient care and treatment in hospitals falls on the shoulders of nurses [11]. Workload refers to the total work done by an individual or team within a specific period. Although the workload is a concept that refers to the number of primary tasks assigned, it can threaten the physical and psychological safety of nurses and reduce job satisfaction while increasing job burnout [12]. Trait anxiety, psychological health, and social isolation are the primary factors affecting Turkish nurses’ quality of life during the COVID-19 pandemic [13]. Therefore, paying close attention to the factors influencing nurses’ performance and workplace, especially in critical situations, is crucial.

There are many consequences and preoccupations brought about by COVID-19, such as the severity of the disease, its unpredictability, and the lack of knowledge about the timing of the disease’s outbreak [14]. Fear and anxiety about possible infection with COVID-19 are destructive, as they can cause stress and psychological abnormalities in individuals [15]. The nature of this disease increases severe stress reactions, such as fatigue, anxiety, and depression in nurses [16]. A study indicated that among healthcare workers, nurses experienced higher anxiety about infection with COVID-19 for themselves and their families [9]. Evidence and data indicate that nursing care for COVID-19 patients is challenging and exhausting, and a high volume of services and work shift restrictions make nurses exhausted. Nurses participating in a study mentioned that patients’ higher care needs and fewer nursing personnel increased the nurses’ workload and fatigue [17]. The COVID-19 epidemic and changes in work status have significantly impacted nurses’ lives personally and professionally. In today’s interconnected world, the integration of personal and work life has resulted in work life overshadowing personal life, leading to the emergence of the quality of work life (QWL) [18]. QWL refers to the satisfaction of workers with their personal and work-related needs within their job roles [19]. Unlike in the past, where the focus was primarily on personal life, improving work life has now become a crucial social issue worldwide, with organizations and employees striving to achieve this goal [20].

QWL encompasses workplace processes, strategies, and conditions that contribute to employees’ overall job satisfaction, which, in turn, relies on favorable work conditions and organizational efficiency [21]. In order to enhance and optimize organizational efficiency, prioritizing employees’ capabilities, physical and mental health, and performance is essential [22]. The QWL and practical job performance have been recognized as critical success factors for any organization, including healthcare institutions like hospitals since 1973 [23].

By serving as an index, QWL offers valuable insights to managers regarding employees’ primary concerns, fostering a sense of ownership, self-management, security, and responsibility, thereby increasing employee productivity [24]. QWL has a direct relationship with job satisfaction but an inverse relationship with job turnover [25]. Therefore, QWL is essential in improving organizational commitment among healthcare workers [26], nurses’ performance, and care performance and outcomes [4].

Health managers, especially hospital managers, must take appropriate measures to make necessary changes in the model and contents of academic education in response to experiences related to recent events or other care conditions, such as natural crises [27], as well as management considerations. Undoubtedly, evaluating the workload and QWL of nurses is essential, despite the effective measures taken by health managers, particularly hospital managers, to recruit a new workforce, balance nurse workload, and provide suitable facilities and incentives.

The present study aimed to answer the question, “What is the relationship between the workload and the QWL of nurses providing care for patients with diseases?” Given the heterogeneous findings about workload [28] and the nurses’ QWL before the COVID-19 pandemic [29], the present study was thus conducted to determine: (1) the relationship between workload and the QWL of nurses caring for COVID-19 patients admitted to Imam Hossein Hospital, affiliated with Shahrud University of Medical Sciences, and (2) to elucidate the factors predicting their QWL.

## Methods

### Design, setting, and participants

The present cross-sectional study was conducted from October 10, 2021, to January 15, 2022. The city of Shahrud has three hospitals, two of which are affiliated with the university of medical sciences. Among these three hospitals, only one, Imam Hossein Hospital, serves as the designated referral center for COVID-19 patients. It has dedicated emergency units, infectious disease wards, and ICUs for their admission. In the sample, we included 250 nurses caring for COVID-19 patients admitted to Imam Hossein Hospital of Shahrud. We applied the inclusion and exclusion criteria using census sampling to select all nurses for the study.

The inclusion criteria were having at least three months of work experience caring for COVID-19 patients and expressing willingness and consent to participate in the study. We considered returning incomplete questionnaires and unwillingness to participate in research as the exclusion criteria.

### Ethical considerations

Data collection began after the hospital management approved the project and obtained ethical approval (Approval: IR.SEMUMS.REC.1400.282). During the rest time of these nurses in a work shifts, the researcher explained the research objective to them and assured them that the research findings would be used only for research purposes and would be anonymous and confidential. The participating nurses completed informed consent forms to participate in the study and returned the questionnaires afterward.

### Instruments and data collection

We collected data using the researcher-made demographic information questionnaire, which included questions about age, gender, marital status, clinical work experience, work shifts, and work experience in the COVID-19 unit. The NASA Task Load Index (TLX) questionnaire and Walton’s Quality of Work Life (QWL) questionnaire were employed.

The NASA Task Load Index questionnaire consists of two sections. The first section classifies the total activity workload into six subscales: Mental demand, Physical demand, Temporal demand, Overall performance, Effort, and Frustration. Each subscale ranks on a 100-point scale with 5-point steps. Individuals establish personal weights based on their perceived importance through pairwise comparisons. They then multiply these weights by the scale score of each dimension, divide them by 15, and obtain a workload score ranging from 0 to 100, representing the total workload index. Mean scores below 50 are acceptable, while scores above 50 indicate a high workload [30]. The reliability coefficient of the NASA-TLX scale has been reported as 0.746 using the test-retest method [31]. Additionally, the questionnaire’s reliability was confirmed among 30 nurses, yielding a Cronbach’s alpha of 0.847 [32]. In this study, we confirmed the reliability of the NASA-TLX through a pilot study involving ten nurses, resulting in a Cronbach’s alpha of 0.89.

Walton’s QWL questionnaire (1973) encompasses components such as Adequate and fair compensations, Safety and health in working conditions, Work and total living space, Constitutionalism in the organization of work, Career opportunities and job security, Opportunity to use and develop human capabilities, Social relevance of work life, and Social integration in the organization [33]. The questionnaire includes 35 closed-ended questions, categorized on a 5-point Likert scale. The total score of each field and all questions determines the QWL index, which ranges from a minimum of 35 to a maximum of 175. Higher scores indicate better QWL. Previous studies among Iranian hospital workers and nurses have investigated the reliability and validity of this tool, confirming its validity and reporting a reliability of 0.94 using the Cronbach’s alpha test [34]. In our pilot study involving ten nurses, we confirmed the reliability of the QWL questionnaire, which yielded a Cronbach’s alpha of 0.80.

### Data analysis

We performed statistical analysis using SPSS-26 software at a significance level of 0.05. We used descriptive and inferential statistics to analyze the data. We described, classified, and compared the research data using relative and absolute frequency tables. Before analyzing the data, we used the Kolmogorov-Smirnov test to determine the normal distribution. We analyzed the collected data using an independent t-test, Pearson’s correlation coefficient, and one-way analysis of variance (ANOVA). Additionally, we utilized backward multiple linear regression analysis to examine the prediction role of workload subscales and demographic characteristics in nurses’ QWL during the COVID-19 pandemic.

## Results

### Demographic characteristics of the participants

The nurses’ mean age was 32.92 years, 73.6% were females, and 69.6% were married. The nurses’ mean work experience was 8.75 years, and 15 months were related to working in COVID-19 care units. Furthermore, 92% of nurses were working in rotational shifts (Table 1).

**Table 1:** Demographic characteristics of the participants

	Frequency	Percentage
**Gender**		
Male	66	26.4
Female	184	73.6
**Marital status**		
Single	76	30.4
Married	174	69.6
**Work shift**		
Morning	11	4.4
Night	8	3.2
Rotational	230	92
	**Mean**	**SD**
**Age**	32.92	6.74
**Work experience (Year)**	8.75	6.41
**Work experience in COVID-19 unit (month)**	15.04	7.64

### The mean score of workload, Walton’s quality of work life, and their subscales

Based on the results, the participants’ mean workload and QWL were 71.43 ± 14.15 and 88.26 ± 19.50, respectively. The maximum perceived workload belonged to physical demand (14.82 ± 8.27) and mental demand (14.36 ± 7.43), and the minimum workload belonged to overall performance (6.63 ± 6.31). The maximum score of QWL subscales belonged to safety and health in working conditions (15.46 ± 4.11), and the minimum score was related to the work and total living space (6.52 ± 2.47) (Table 2).

**Table 2:** Mean score of Workload, Walton’s Quality of Work Life, and their Subscales

Variable	Mean	SD
**Workload**		
Mental demand	14.36	7.43
Physical demand	14.82	8.27
Temporal demand	13.59	7.24
Overall performance	6.63	6.31
Effort	11.78	6.84
Frustration	10.24	9.23
Total workload	71.43	14.15
**Quality of work life (QWL)**		
Social relevance of work life	13.81	4.01
Work and total living space	6.52	2.47
Constitutionalism in the organization of work	9.59	2.91
Social integration in the organization	11.76	3.17
Career opportunities and job security	9.38	2.65
Opportunity to use and develop human capabilities	14.52	3.84
Safety and health in working conditions	15.46	4.11
Adequate and fair compensations	7.16	2.38
Total QWL mean score	88.26	19.50

*SD* Standard deviation

### Relationship between workload, the quality of work life, and demographic variables in nurses

The study of the relationship between workload and its subscales with demographic variables indicated that workload had a positive and significant correlation only with age (`*r* = 0.140, *p* = 0.027). Even though there was no difference between the workload of single and married nurses, there was a negative and significant relationship between marital status and mental demand (*r*=-0.126, *p* = 0.046). Furthermore, there was a significant positive relationship between the number of children and temporal demand (*r* = 0.155, *p* = 0.014). There was no statistically significant relationship between work experience and workload (*r* = 0.096, *p* = 0.129) and no significant relationship between workload and work experience in the COVID-19 department (*r* = 0.036, *p* = 0.568). However, a statistically significant relationship existed between work experience and temporal demand (*r* = 0.179, *p* = 0.004).

The study of the relationship between QWL and its subscales with demographic variables indicated no statistical association between age and QWL (*p* = 0.057). There was a strong positive association between age and fair compensations for the subscales of QWL (*r* = 0.151, *p* = 0.014). Pearson’s test showed no significant relationship between marital status and the number of children with a nurse’s QWL (*p* = 0.618 and *p* = 0.311, respectively). Regarding the subscales of the QWL, there was a significant positive correlation between fair compensations and the number of children (*r* = 0.155, *p* = 0.014).

Furthermore, there was no significant statistical relationship between the QWL, work experience (*r*=-0.078, *p* = 0.222), and work experience in the COVID-19 unit (*r* = 0.006, *p* = 0.923), but there was a negative and significant relationship between fair compensations subscale and work experience (*r* =-0.159, *p* = 0.016).

### Relationship between workload, the quality of work life, and work shift in nurses

Even though the workload of night shifts was less than in the morning and afternoon, the results of the one-way analysis of variance test (ANOVA) indicated no statistically significant relationship between workload and shift work, and it was the same for the relationship between each workload subscale and work shift. The frustration score on the night shift was close to the significance level (*p* = 0.059). Also, the results of One-way ANOVA indicated no significant difference between the nurses’ QWL in different shifts. There was no significant relationship between work shifts and QWL (*p* = 0.933), but the nurses’ QWL was more appropriate in night shifts (Table 3).

**Table 3:** Relationship between Workload, the Quality of Work Life and Work Shift in Nurses

Work shift	Morning	Night	Rotational	F	*P*-value^a^
	Mean (SD)	Mean (SD)	Mean (SD)		
Workload	73.51 (19.81)	68.67 (14.06)	71.45 (13.92)	0.269	0.764
QWL	87.64 (25.84)	92.38 (24.92)	88.04 (19.02)	0.194	0.823

*One-way analysis of variance test (ANOVA)

### The relationship between QWL and its subscales with the workload in nurses

Based on the results, there was a significant negative correlation of 0.308 between workload and QWL in nurses (*r*=-0.308, p < 0.001); in other words, a higher workload decreased the QWL. Furthermore, the workload had a significant inverse relationship with all subscales of the QWL (Table 4).

**Table 4:** The relationship between QWL and its Subscales with the Workload in Nurses

	Workload	
Quality of work life (QWL) subscales	*r*^a^	*P*-value**
Social relevance of work life	-0.255	< 0.001
Work and total living space	-0.286	< 0.001
Constitutionalism in the organization of work	-0.168	0.008
Social integration in the organization	-0.181	0.004
Career opportunities and job security	-0.251	< 0.001
Opportunity to use and develop human capabilities	-0.217	0.001
Safety and health in working conditions	-0.290	< 0.001
Adequate and fair compensations	-0.226	< 0.001
QWL (overall)	-0.308	< 0.001

^a^Pearson’s correlation coefficient

**Correlation is significant at the 0.01 level (2-tailed)

### The multivariate linear regression analysis of the effect of the demographic characteristics and workload subscales on the QWL`

A multiple linear regression model was used to investigate the predictor variables (the demographic characteristics and workload subscales) that had a significant effect on global QWL based on the backward model. To evaluate the extent of the correlation of QWL score with each predictive variable, we used backward linear regression and the ‘margins’ post- estimation command to obtain estimated marginal means and associated confidence intervals. The Kolmogorov–Smirnov test was used to test predictive variables for multicollinearity and the residuals for normal distribution. The results of the regression indicated the four predictors explained 13% of the variance (*R*2 = 0.14, *F*(4,244) = 10.28, *p* < 0.001). It was found that number of children (β = 4.61, *p* = 0.004), work experience (β= − 0.54, *p* = 0.019), Effort (β = 0.37, *p* = 0.033), and total workload (β = −0.44, *p* = 0.000) significantly predicted nurses’ QWL (Table 5).

**Table 5:** The multivariate Linear Regression Analysis of the Effect of the Demographic Characteristics and Workload Subscales on the QWL

Variables	B	Standard Error	Standardized Coefficients	t	*P*-Value	95.0% CI for Beta		Collinearity Statistics	
			Beta			Lower bound	Upper bound	Tolerance	VIF
Number of Children	4.61	1.59	0.22	2.89	0.004	1.48	7.75	0.61	1.62
Work Experience	- 0.54	0.23	- 0.18	- 2.35	0.019	- 0.99	- 0.08	0.60	1.64
Workload: Effort Subscale	0.37	0.17	0.13	2.14	0.033	0.03	0.70	0.96	1.04
Total Workload	- 0.44	0.08	- 0.32	- 5.28	0.000	- 0.60	- 0.27	0.95	1.05
Constant	116.75	6.08		19.17	0.000	104.76	128.74		

R=0.38, R Square=0.14, Adjusted R Square=0.13, Durbin-Watson=1.96

## Discussion

The current study’s findings, which sought to ascertain the relationship between nurses’ workload and the quality of their working lives while caring for COVID-19 patients, showed that the average workload for the nurses was 71.43 ± 14.15. Consistent with the results of the present study, Pourteimour et al. (2021) reported the mean workload of nurses who took care of patients with COVID-19 in Urmia and Hamedan hospitals (67.30 ± 14.53) [35]. The results of studies by Shoja et al. (2020) and Judek et al. (2018) also confirmed this finding [36, 37]. Based on findings of research by Bakhshi et al. in Kermanshah hospitals (2017), the mean ± standard deviation of workload score was 69.73 ± 15.26 [5], and it was 59.95 ± 16.41in a study by Jarahian et al. [6], and it was partially less than the present study. In similar studies, nurses’ mean workload was moderate to low in non-critical situations [38–40]. Malekpour et al. (2014) reported that nurses were responsible for 80% of tasks in health and medical centers and generally had a heavy workload [3]. The COVID-19 epidemic has significantly increased the workload for nurses, as indicated by the results. According to the findings, mental demand was identified as the dimension with the highest perceived workload, while overall performance had the lowest workload, aligning with a study by Gharagozlou et al. (2020) [41]. Shoja et al. (2020) examined the subscales of the workload questionnaire and observed increased scores for mental demand, physical demand, temporal demand, and frustration, leading them to conclude that the COVID-19 pandemic negatively affected staff workload and mental health [36]. A study conducted by Bakhshi et al. (2017) found that mental workload made the highest contribution, while the feeling of frustration had the lowest contribution [5]. Rafiee et al. (2015) conducted a study to measure the mental workload of nurses in the emergency department of a hospital and reported that the dimension of overall performance had the lowest score, while frustration had the highest score [42].

Furthermore, the nurses caring for COVID-19 patients endure high physical and mental demands. Based on the results of some studies conducted before the prevalence of COVID-19, nurses who took care of COVID-19 patients felt more frustrated and discouraged but considered their activities more effective. High and frequent workloads are two key factors leading to exhaustion and burnout [12], resulting in lower overall performance, memory, and thinking process, irritability, annoyance, and reduced learning [43]. The findings of Mohamadzadeh Tabrizis’ study show the negative impact of caring for COVID-19 patients on nurses’ quality of life [44].

This study showed that other than age, there was no link between workload and demographic factors like sex, marital status, number of children, working shifts, and years of work experience. There was a relationship between marital status, mental demand, the number of children, and temporal demand. There was no correlation between workload and work experience in the COVID-19 unit and shift work. Jarahian et al. (2018) found a correlation between workload, work experience, and the number of children. The study indicated that individuals with lower work experience and no children had lower mental workloads [6].

The findings of a study on determinants of workload indicated a significant relationship between physical workload with work experience, age, work pattern, number of shifts, and type of employment, and between temporal demand with body mass index (BMI), work experience, and type of employment [5]. Based on research by Shoja et al., the type of job, work shift, education level, and exposure to COVID-19 affected the workload score [36]. Older nurses, married individuals, and those with children might experience a higher workload due to increased work shifts and forced overtime. The higher workload, particularly regarding temporal demand, is especially evident. Establishing a balance between work life and personal/family demands is vital since research indicates that personal and professional lives often intertwine [4]. Nurses, regardless of gender or age, must work various shifts and wear protective masks in hospitals during the COVID-19 outbreak. Huang et al. (2018) confirm that nurses face high responsibility, heavy workload, intense work pressure, and the need to work in rotational shifts due to the unique nature of their profession [45].

Based on the results, the mean QWL of the nurses was 88.26 ± 19.50. In line with the findings of the present study, Nikeghbal et al. reported that the QWL of nurses who took care of patients with COVID-19 was 92.57 with a standard deviation of 13.2, which was better than the non-COVID-19 caregivers (79.43). A significant association between the two groups was revealed by the comparison (*p* = 0.001) [46]. The results of Mohammadi’s study in Iran [29], and most studies in the world, show that nursing QWL is mainly at a moderate level and requires improvement interventions [40, 42, 47]. Some studies show an increase in depression, anxiety [48], stress, and burnout [49, 50] among nurses during the pandemic of Coronavirus Disease.

Based on the results, the highest score of subscales belonged to safety and health in working conditions, while the lowest score belonged to work and total living space. Like Aminizadeh’s finding in pre-hospital staffs, Opportunity to use and develop human capabilities had a significant role in nurses’ QWL [26]. According to research on the relationship between job burnout, performance, and QWL, the constitutionalism in how work is organized contributed the most to the QWL score, while the social significance of work life contributed the least [51].

In a study on the relationship between the components of the QWL and job satisfaction of midwives, the results indicated that providing career opportunities and job security had the most significant contribution, and social relevance of work life in the organization had the minor contribution to the QWL in the midwives [52].

Falahi Khoshknab’s study before the COVID-19 pandemic indicated that 21% of nurses described their quality of work life (QWL) as moderate, while 67% described it as good, and 11% of nurses were delighted with their QWL [53]. In a study by Faraji et al. conducted prior to the prevalence of COVID-19, 61% of nurses had a low QWL, and even 39.7% of them wanted to leave their jobs [54]. In line with these studies, the current study, as well as Jafari’s study [48], suggests that nurses must receive adequate support to overcome workplace stressors. The findings of Shirali’s study showed job stress and low resilience as threatening factors in nurses during the care of COVID-19 patients [55]. Therefore, support for nurses should focus on both individual and organizational aspects.

Nurses’ workload has significantly increased, yet their work-life quality remains moderate. Various factors contribute to this category, and we will address some below. The present study identified a significant relationship among age, number of children, work experience, and specific aspects of QWL. Previous studies, such as those conducted by Gharagozlou et al. and Shafipour et al., found no significant relationship between QWL and demographic variables like age, gender, and marital status [41, 56]. Dehghannayieri et al. [57] and Dargahi et al. [58] also found no significant relationship between marital status and QWL, but Khaghanizadeh [59] and Falahi Khoshknab [53] reported a significant relationship. Mohammadi et al. (2017) reported a significant correlation between the QWL and employment status, shift work, hospital, and satisfaction with the field of study (*p* < 0.001) [29]. According to Gharagozlou’s study, there was no significant relationship between nurses’ QWL with the number of shifts and the number of patients in each shift, but there was a significant relationship between the QWL and overtime hours among the nurses [41]. In a study by Shafipour et al., there were significant relationships between the QWL, overtime hours, number of night shifts per month, and income level, but there was no significant relationship with the job unit [56]. A study indicated that working on the night shift negatively influences nurses’ QWL [60]. Researchers considered possible reasons for contradictions and differences in the types of sectors based on the characteristics of the participants and the study time (before and during the pandemic) in the study. The present study reported no difference among different shifts regarding QWL, but the night shift was associated with a better QWL. Researchers believe this was because most diagnostic and therapeutic procedures were carried out during the morning shift, and systemic supervision and nurses’ freedom of action were reduced during the night shift. However, the difference was not statistically significant.

Furthermore, there was a relationship between work experience and adequate and fair compensation, indicating the financial concerns of married nurses with higher experience and more children. In the present study, the Opportunity to use and develop human capabilities and safety and health in working conditions had the most significant contribution, adequate and fair compensations, and work and total living space had the minor contribution. Consistent with this finding, Jafari et al. reported that the highest contribution to the QWL was the development of human capabilities, career opportunities, and job security, but the lowest was fair compensations [34]. In a study by Dargahi et al. [58] and research in Ethiopia [4], there was a significant relationship between the monthly income level and the QWL. The QWL increased with higher total compensation. A study in Canada indicated that a higher level of income increased the QWL [61], but in a study by Nikeghbal et al., there was a significant relationship between the QWL index (adequate and fair compensations) and monthly income only in nurses who took care of COVID-19 patients [46].

The results of this study indicate a significant inverse relationship between nurses’ workload and quality of work life (QWL). In other words, a higher workload leads to a decrease in QWL. The COVID-19 pandemic has imposed difficult circumstances, and due to the intensity of the workload, nurses have had to put their own lives and the lives of their loved ones at risk while treating COVID-19 patients. The risk of infection and death from COVID-19 has caused significant psychosocial stress for nurses and other healthcare professionals [62]. In similar studies, Lai et al. [63] and Gharagozlou et al. [41] investigated the QWL and workload status using the same tools as in the present study and reported significant relationships between different dimensions of workload and QWL.

Numerous studies have indicated that a high workload endangers the quality and safety of patient care, increases errors, and ultimately prolongs hospitalization time. This situation affects the relationship between nurses, physicians, and patients [64]. In a study by Ardesatni Rostami et al. (2019) focusing on nurses in ICUs, a negative correlation was found between workload and job performance. According to the study, 75% of nurses rated their performance moderate [65]. These studies were conducted primarily before the COVID-19 pandemic, but nurses have always had to work long hours to manage their workload. Although the pandemic has further increased their workload, it has not significantly impacted the quality of their work life. Similar to the pre-COVID-19 era, the quality of work life remains predominantly dependent on organizational factors such as support and financial assistance.

The present findings indicate that an increase in the number of children and effort contributes to a high level of QWL, while work experience and total workload decrease the QWL of nurses during the COVID-19 pandemic. Consistent with these findings, Navales et al. (2021) reported a relationship between individual factors, such as older nurses, females, bachelor graduates with more dependents, more children, and job positions with more extended work experience, and nurses’ QWL in Indonesia [66]. Woon et al. (2021) found that social support from friends and significant others (such as children and spouse) predicted higher QWL. Despite the COVID-19 restrictions, encouragement regarding family and children appears to have positively influenced the quality of nurses’ work life [67].

Contrary to the findings of Gharagozlou’s study, nurses who encounter COVID-19 patients require additional effort, potentially resulting in an enhanced QWL [41]. Hence, despite the numerous adverse effects of the COVID-19 pandemic on nurses, it has also presented opportunities for provisional, organizational, and individual improvements in their quality of life.

The mental burden experienced by nurses in these job groups is significant. Several factors contribute to the creation and escalation of this burden, including consistent and uninterrupted work, work duration, job requirements (such as concentration, accuracy, and effort), physical stress-induced fatigue, age, work experience, environmental factors (such as sound and vibration), equipment usage, individual feedback on work and interpersonal interactions, overtime, and ergonomic working conditions [46]. Thus, these factors are among those that contribute to the increased workload of nurses. Furthermore, as these employees operate within a consistent and stable work environment characterized by the nature of the job and working conditions, they often have longer working hours. Consequently, this leads to physical and mental fatigue, exhaustion, burnout and ultimately diminishing their QWL. In line with the negative effect of workload on QWL, Nikeghbal et al.‘s study supported that an increased workload was associated with a decreased QWL [46].

### Limitations of the study

Since we conducted this research solely on the Shahrud University of Medical Sciences nursing staff, it is essential to exercise caution when generalizing the results to other settings. We recommend conducting multicenter studies with larger sample sizes. We maintained a continuous presence in different units and shifts to address the main barriers for nurses to participate in the study, namely the lack of free time and high workload. We also followed up to increase participation in the study. Another limitation was that nurses were less focused on answering the questionnaires because they had to complete them during work hours. Some nurses hesitated to complete the questionnaires due to insufficient information from prior research. We assured the participants that they would have access to the research results. Furthermore, it is worth noting that the participants’ mental state during the questionnaire completion could also influence the research results.

### Recommendations for future research

We recommend conducting multicenter studies with larger samples in settings with different cultures to identify other unrecognized effective factors in relation to workload and QWL. It is essential to carry out interventional studies, mainly focusing on nurses’ psychological empowerment and organizational support. Additionally, qualitative research is required to explain the process of QWL development and comprehend nurses’ lived experiences.

### Clinical implications for nursing managers and policymakers

In order to achieve a better quality of work life (QWL), nursing managers can take active steps to improve nurses’ work conditions. These steps include reducing nurses’ workload, creating a respectful working atmosphere, considering their work experience, work shifts, and age, and ensuring adequate and fair pay. Additionally, effective measures should be taken to recruit a new workforce, balance nurses’ workload, and provide suitable facilities and incentives.

## Conclusion

The high workload was a significant stressor for hospital staff and nurses. The nurses’ workload increased, leading to increased stress and decreased productivity, ultimately affecting their quality of life. Compared to studies before the outbreak of COVID-19, there was a partial increase in nurses’ workload, but there was no noticeable change in the quality of their work life, which remained at a moderate level. Therefore, acquiring knowledge about the influential factors for positive quality of work life (QWL) development among nurses is crucial to improve their QWL. Developing QWL in nurses can increase their loyalty to the profession and promote the quality of care and patient satisfaction while decreasing nurses’ exhaustion and burnout.

## Data Availability

All the data supporting the study findings are within the manuscript. Additional detailed information and raw data are available from the corresponding author upon reasonable request.

## Abbreviations

WHO: World Health Organization
QWL: Quality of work life
